# Ganglion cell layer thinning in diabetic patients without retinopathy: related or unrelated to total macular thickness?

**DOI:** 10.5935/0004-2749.20200048

**Published:** 2024-02-11

**Authors:** Selma A. Somilleda-Ventura, Dulce M. Razo Blanco-Hernández, Itzel Ocampo-Moreno, Virgilio Lima-Gómez

**Affiliations:** 1 Research Direction, Hospital Juárez de México, Ciudad de México, México; 2 Research Division, Hospital Juárez de México, Ciudad de México, México; 3 Ophthalmology service, Hospital Juárez de México, Ciudad de México, México

**Keywords:** Diabetes Mellitus, Retinal ganglion cells, Retinal vessels, Fluorescein angiography, Macula lutea, Tomography, optical coherence, Tonometry, ocular, Diabetes Mellitus, Células ganglionares da retina, Vasos retinianos, Angiofluoresceínografia, Mácula lútea, Tomografia de coerência óptica, Tonometria ocular

## Abstract

**Purpose:**

Reduction of ganglion cell layer thickness may occur in diabetic patients without retinopathy. The relationships of this preclinical finding with retinal thickness or reduced parafoveal vessel density have not been established. This study investigated the relationships of ganglion cell layer thickness with retinal thickness and parafoveal vessel density in patients with and without diabetes.

**Methods:**

This was an observational, cross-sectional, prospective study that used optical coherence tomography angiography to compare non-diabetic patients (group 1) with diabetic patients without retinopathy (group 2). Ganglion cell layer thickness, macular thickness, and parafoveal vessel density (central, inner, and complete) medians were compared between groups (Mann-Whitney U test), and their relationships were assessed in each group (Spearman Rho test).

**Results:**

In total, 68 eyes were included in this study: 34 in group 1 and 34 in group 2. Ganglion cell layer thickness did not differ between groups in any sector. There were strong positive correlations between fields 2 (superior parafoveal), 3 (temporal parafoveal), and 4 (inferior parafoveal) of the optical coherence tomography macular thickness map and the ganglion cell layer thickness in all sectors in both groups. Central vessel density mean was lower in diabetic patients. In group 1 alone, thickness changes in the inferior and nasal inferior ganglion cell layer sectors were partially explained by inner vessel density (r^2^=0.32 and r^2^=0.27).

**Conclusions:**

Mean ganglion cell layer thickness was not lower in diabetic patients without retinopathy than in non-diabetic patients. Moreover, it exhibited a substantial correlation with total macular thickness. Parafoveal vessel density decreased before ganglion cell layer thinning was observed.

## Abstract (pt)

**Objetivo:**

Pode ocorrer redução da espessura da camada de células ganglionares em pacientes diabéticos sem retinopatia. As relações desse achado pré-clínico com a espessura da retina ou a densidade reduzida de vasos parafoveais não foram estabelecidas. Este estudo investigou as relações da espessura da camada de células ganglionares com a espessura da retina e densidade dos vasos parafoveais em pacientes com e sem diabetes.

**Métodos:**

Estudo prospectivo, observacional, transversal que utilizou angiotomografia de coerência óptica para comparar pacientes não diabéticos (grupo 1) com pacientes diabéticos sem retinopatia (grupo 2). As médias da espessura da camada de células ganglionares, espessura macular e densidade dos vasos parafoveais (central, interno e completo) foram comparadas entre os grupos (teste U de Mann-Whitney) e suas relações foram avaliadas em cada grupo (Teste de Spearman Rho).

**Resultados:**

No total, 68 olhos foram incluídos neste estudo: 34 no grupo 1 e 34 no grupo 2. A espessura da camada de células ganglionares não diferiu entre os grupos em nenhum setor. Houve fortes correlações positivas entre os campos 2 (parafoveal superior), 3 (parafoveal temporal) e 4 (parafoveal inferior) do mapa da espessura macular da tomografia de coerência óptica e a espessura da camada de células ganglionares em todos os setores dos dois grupos. A média da densidade central dos vasos foi menor nos pacientes diabéticos. Somente no grupo 1, as alterações de espessura da camada de células ganglionares nos setores inferior e nasal inferior foram parcialmente explicadas pela densidade do vaso interno (r^2^=0,32 e r^2^=0,27).

**Conclusões:**

A média da espessura da camada de células ganglionares não foi menor em pacientes diabéticos sem retinopatia do que em pacientes não diabéticos. Além disso, exibiu uma correlação substancial com a espessura macular total. A densidade dos vasos parafoveais diminui antes do desbaste da camada de células ganglionares.

## INTRODUCTION

Diabetic retinopathy is a chronic and specific complication of diabetes, with clinical manifestations that result from microvascular injury^(1)^. Damage to retinal neurons may occur in patients with this disease, which presumably precedes ophthalmoscopic changes^(2)^. Neural damage could explain the reduction of foveal sensitivity (measured by automated perimetry) that occurs in diabetic patients without retinopathy^(3)^. Diabetes affects the ganglion cell layer (GCL) of the retina, which can be measured with optical coherence tomography (OCT)^(4)^. OCT can be used to quantify the thickness of the entire retina, and currently available spectral domain equipment can selectively measure the thickness of the GCL. The OCT equipment also possesses an angiography function (OCT angiography, OCTA) that allows measurement of parafoveal vessel density^(5)^. Both GCL thickness and parafoveal vessel density may decrease in diabetic patients before the appearance of clinical signs of diabetic retinopathy. These changes indicate neural and vascular preclinical damage^(6)^, respectively.

Reduction of GCL thickness has been described in diabetic patients without retinopathy. In addition, similar changes have been observed involving total pericentral macular thickness in diabetic patients with minimal retinopathy^(7)^ and foveal center thickness in diabetic patients without retinopathy^(8)^. Although the findings in those studies suggested that low total macular thickness values could be the result of neural loss, the investigators did not measure GCL thickness. Thus, it is unknown whether both thicknesses exhibit relationships that could predict such changes before the appearance of clinical retinopathy. Parafoveal vessel density may decrease in diabetic patients without retinopathy^(9)^ and could serve as a reference variable when determining whether the reduction of GCL thickness precedes vascular preclinical retinal changes in diabetes. This study was conducted to compare GCL thickness between non-diabetic patients and diabetic patients without diabetic retinopathy, to evaluate the correlation between GCL thickness and total macular thickness, and to determine whether GCL thinning is present before the reduction of parafoveal vessel density, which is already described in our population.

## METHODS

### Patients

This was an observational, cross-sectional, comparative, prospective study. The study population was recruited from among patients attending a federal reference hospital in México City, México, during the period from January 6 to March 31, 2018. The study protocol adhered to the tenets of the Declaration of Helsinki and was approved by the Institutional Review Board of the hospital where it was performed.

The study included patients aged 40-70 years of any sex. The inclusion criteria were as follows: 1) patients had no diabetes or had type 2 diabetes mellitus without diabetic retinopathy, 2) patients had ocular media that allowed collection of an optical coherence tomography thickness map and an OCTA map, 3) patients signed informed consent to participate in the study, and 4) patients had no other retinopathy, no previous intraocular surgery, and no treatment with anti-inflammatory drugs or diuretics. The elimination criteria were withdrawal of informed consent, the presence of any sign of diabetic retinopathy in fundus photographs, and/or a foveal avascular zone (FAZ) diameter >0.92 mm. This diameter was more than two standard deviations greater than the average found in patients with diabetic retinopathy^(9)^ and was considered suggestive of ischemia.

### Measurements

Evaluations in all patients included measurement of best-corrected visual acuity in decimal equivalents and a 45° fundus photograph. A single researcher obtained macular maps using OCTA Cirrus 5000 HD with Angioplex equipment (Zeiss Meditec, Dublin, CA, USA) in the following manner: 1) a 6 × 6-mm macular cube of retinal thickness, 2) a 3 × 3-mm angiography scan via autofluorescence analysis of the superficial vascular plexus using the automatic segmentation algorithm of the equipment, and 3) a 152 × 128-mm macular map for the analysis of GCL thickness. All maps were confirmed to have correct centering of the fovea, and there were no image artifacts (eyelashes, movement, or any other artifact) that blocked the OCTA signal; only images with a signal strength >7 were used.

The variables assessed in each patient by using OCTA measurements were as follows: retinal thickness in the nine fields of the macular map; macular volume; parafoveal vessel density in the superficial capillary plexus (central: 1 mm concentric to the fovea; inner: within 0.5 to 1.5 mm from the foveal center; complete: the entire 3-mm diameter region concentric to the fovea); area and diameter of the FAZ; GCL thickness in six sectors: superior (S), nasal superior (NS), temporal superior (TS), inferior (I), nasal inferior (NI), and temporal inferior (TI); and retinal nerve fiber layer (RNFL) thickness. All OCT thicknesses and OCTA measurements were generated automatically by the equipment.

### Outcome variables

Non-diabetic patients were assigned to group 1, and diabetic patients were assigned to group 2. The main outcome variable was the GCL thickness in each sector. Retinal thicknesses in fields 2 (superior parafoveal), 3 (temporal parafoveal), 4 (inferior parafoveal), and 5 (nasal parafoveal) of the macular map were regarded as secondary variables because they corresponded to the regions of GCL measurements. Parafoveal vessel density was measured as a reference variable.

### Statistical analysis

The median values of retinal thickness, parafoveal vessel density, and GCL thickness were compared between groups using the Mann-Whitney U test. Relationships of retinal thickness in fields 2, 3, 4, and 5 of the macular map with parafoveal vessel density and GCL thickness were determined using the Spearman Rho test. A p value <0.05 was considered significant. Statistical analyzes were performed using SPSS for Windows (version 22, IBM Corp., Armonk, NY, USA).

## RESULTS

We evaluated 68 eyes of 39 patients (34 eyes per group). The mean ± standard deviation (SD) age was 55.08 ± 9.17 years, and 17 patients (52.6%) were women. The diabetes duration in group 2 ranged from 0.01 to 17 years (mean ± SD, 7.08 ± 5.22), and 33 patients in group 2 (94.2%) received only oral hypoglycemic agents. Visual acuity among all patients ranged from 0.33 to 1.00 in decimal equivalents (mean ± SD, 0.84 ± 0.21).

Table 1 presents the GCL thickness, macular thickness, and vessel density in both groups. Although median GCL thickness was larger in the S, ST, I, NS, and NI sectors in diabetic patients, GCL thickness did not significantly differ between groups in any sector. Central vessel density was significantly lower in group 2 than in group 1.

**Table 1:** Comparisons of GCL sectors, retinal thicknesses, and parafoveal vessel densities between groups (median, interquartile rank)

Variable	Group 1 (n=34)	Group 2 (n=34)	p^*^
Superior (µm)	79.00, 6.75	82.00, 10.00	0.26
Superior temporal (µm)	79.00, 8.75	80.00, 5.25	0.96
Temporal inferior (µm)	79.00, 10.75	79.00, 8.00	0.90
Inferior (µm)	77.50, 13.50	79.00, 6.25	0.72
Nasal inferior (µm)	79.50, 12.75	81.50, 10.25	0.53
Nasal superior (µm)	80.00, 10.50	84.00, 9.75	0.23
Field 2 (µm)	320.50, 21.50	321.50, 25.75	0.06
Field 3 (µm)	308.00, 21.25	308.00, 25.50	0.43
Field 4 (µm)	317.00, 21.00	316.00, 27.75	0.38
Field 5 (µm)	321.50, 24.50	325.00, 22.50	0.001
RNFL	90.00, 15.25	94.00, 11.25	0.33
FAZ area	0.18, 0.14	0.27, 0.16	0.14
FAZ diameter	0.63, 0.29	0.72, 0.20	0.25
Central vessel density	11.10, 4.52	9.05, 3.73	0.02
Inner vessel density	20.60, 4.75	20.90, 1.40	0.52
Complete vessel density	19.50, 4.15	19.65, 1.35	0.36

FAZ= foveal avascular zone; RNFL= retinal nerve fiber layer

* Mann-Whitney U test

**Figure 1:**
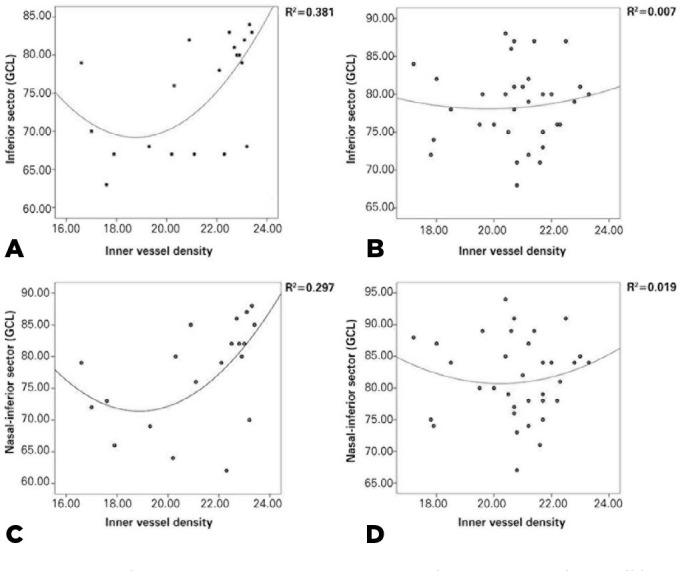
Correlations between inferior and nasal inferior ganglion cell layer (GCL) sectors and inner vessel density in each group. A) and C) correspond to group 1, and B) and D) correspond to group 2.

In both groups, there were strong positive correlations between macular thickness map fields (2, 3, and 4) and GCL thickness in all six sectors (Table 2). In group 2, this correlation was also detected in field 5 (Table 3). In group 1 alone, there were positive correlations between the inner vessel density and GCL thickness in sectors S, TI, I, NI, and NS, and there were also positive correlations between complete vessel density and sectors S, I, and NI (Table 4). In group 2, the FAZ diameter was positively correlated with GCL thickness in the TI sector (r=0.56, p=0.01) (Table 5). Changes in GCL thickness in sectors I and NI were partially explained by the inner vessel density (r^2^=0.32 and r^2^=0.27, respectively) in group 1, but these relationships were absent in group 2 (Figure 1).

**Table 2:** Relationships of GCL sectors with retinal thicknesses in group 1

Variable	S		TS		TI		I			NI		NS		Field 2	Field 3	Field 4	Field 5
Field 2	0.61^**^		0.68^**^		0.60^**^		0.47^**^			0.48^**^		0.57^**^		1	0.71^**^	0.63^**^	0.57^**^
Field 3	0.68^**^		0.69^**^		0.68^**^		0.58^**^			0.62^**^		0.60^*^		---	1	0.79^**^	0.02
Field 4	0.57^*^		0.50^*^		0.58^**^		0.47^*^			0.57^**^		0.54^*^		---	---	1	0.19
Field 5	0.09		0.19		0.12		0.02			0.01		0.10		---	---	---	1
S	1		0.80^**^		0.88^**^		0.85^**^			0.82^**^		0.90^**^		---	---	---	---
TS	---		1		0.78^**^		0.66^**^			0.66^**^		0.73^**^		---	---	---	---
TI	---		---		1		0.93^**^			0.81^**^		0.78^**^		---	---	---	---
I	---		---		---		1			0.87^**^		0.78^**^		---	---	---	---
NI	---		---		---		---			1		0.90^**^		---	---	---	---
NS	---		---		---		---			---		1		---	---	---	---

S = superior; TS= temporal superior; TI= temporal inferior; I= inferior; NI= nasal inferior; NS= nasal superior.

* p<0.05, Spearman Rho Test;

** p<0.01, Spearman Rsho Test.

**Table 3:** Relationships of GCL sectors with retinal thicknesses in group 2

Variable		S		TS		TI		I		NI		NS		Field 2	Field 3	Field 4	Field 5
Field 2		0.65**		0.77**		0.72**		0.67**		0.67**		0.63**		1	0.96**	0.93**	0.97**
Field 3		0.58**		0.74**		0.74**		0.69**		0.66**		0.57*		---	1	0.96**	0.94**
Field 4		0.59**		0.71*		0.77**		0.77*		0.70**		0.58**		---	---	1	0.92**
Field 5		0.65**		0.71*		0.71**		0.71*		0.68**		0.63**		---	---	---	1
S		1		0.83**		0.81**		079**		0.80**		0.94**		---	---	---	---
TS		---		1		0.87**		0.76**		0.72**		0.78**		---	---	---	---
TI		---		---		1		0.88**		0.80**		0.81**		---	---	---	---
I		---		---		---		1		0.92**		0.84**		---	---	---	---
NI		---		---		---		---		1		0.89**		---	---	---	---
NS		---		---		---		---		---		1		---	---	---	---

S= superior; TS= temporal superior; TI= temporal inferior; I= inferior; NI= nasal inferior; NS= nasal superior. *p<0.05, Spearman Rho Test; **p<0.01, Spearman Rho Test.

**Table 4:** Relationships of GCL sectors with parafoveal vessel density and FAZ diameter in group 1

Variable		S	TS		TI		I		NI		NS		Central VD		Inner VD	Complete VD	FAZ d
Central VD		0.17	0.18		0.09		0.02		0.01		0.11		1		0.37	0.50^*^	-0.68^**^
Inner VD		0.53^*^	0.36		0.44^*^		0.60^**^		0.58^**^		0.49^*^		---		1	0.97^**^	0.27
Complete VD		0.47^*^	0.31		0.40		0.53^*^		0.50^*^		0.44		---		---	1	0.17
FAZ d		0.011	0.009		0.16		0.28		0.27		0.12		---		---	---	1
S		1	0.80^**^		0.88^**^		0.85^**^		0.82^**^		0.90^**^		---		---	---	---
TS		---	1		0.78^**^		0.66^**^		0.66^**^		0.73^**^		---		---	---	---
TI		---	---		1		0.93^**^		0.81^**^		0.78^**^		---		---	---	---
I		---	---		---		1		0.87^**^		0.78^**^		---		---	---	---
NI		---	---		---		---		1		0.90^**^		---		---	---	---
NS		---	---		---		---		---		1		---		---	---	---

S= superior; TS = temporal superior; TI = temporal inferior; I = inferior; NI = nasal inferior; NS = nasal superior; VD = vessel density; FAZ d = foveal avascular zone diameter.

* p<0.05, Spearman Rho Test;

** p<0.01, Spearman Rho Test.

**Table 5:** Relationships of GCL sectors with parafoveal vessel density and FAZ diameter in group 2

Variable	S	TS	TI	I	NI	NS	Central VD	Inner VD	Complete VD	FAZ d
Central VD	-0.002	-0.20	-0.24	-0.17	-0.14	-0.13	1	0.57^**^	0.71^**^	-0.68^**^
Inner VD	-0.05	-0.15	-0.03	-0.01	-0.02	-0.06	---	1	0.97^**^	-0.42
Complete VD	-0.05	-0.21	-0.10	-0.06	-0.06	-0.08	---	---	1	-0.21
FAZ d	-0.12	-0.05	0.56^**^	-0.10	-0.06	-0.003	---	---	---	1
S	1	0.77^**^	0.85^**^	0.83^**^	0.92^**^	0.91^**^	---	---	---	---
TS	---	1	0.75^**^	0.62^**^	0.65^**^	0.58^**^	---	---	---	---
TI	---	---	1	0.90^**^	0.85^**^	0.70^**^	---	---	---	---
I	---	---	---	1	0.89^**^	0.71^**^	---	---	---	---
NI	---	---	---	---	1	0.91^**^	---	---	---	---
NS	---	---	---	---	---	1	---	---	---	---

VD= vessel density; FAZ d= foveal avascular zone diameter; S= superior; TS= temporal superior; TI= temporal inferior; I= inferior; NI= nasal inferior; NS= nasal superior

* p<0.05, Spearman Rho Test;

** p<0.01, Spearman Rho Test.

## DISCUSSION

GCL thickness did not differ between age-matched diabetic patients without retinopathy and non-diabetic patients, and approximately 50% of changes in GCL thickness exhibited a similar direction to that of total macular thickness in both groups.

Mean GCL thicknesses are typically greater than 80 µm in non-diabetic individuals^(10-12)^, and these measurements are reproducible and decrease by 0.25 µm/year^(12)^. In diabetic patients without retinopathy, compared with non-diabetic patients, the mean GCL thicknesses were reportedly 1.4^(13)^ and 4.37 µm^(14)^ thinner (p>0.05), and one study reported lower mean GCL thicknesses in the NS (p=0.022) and NI (p=0.042) sectors^(15)^. Another study reported reductions of mean GCL in all sectors of the GCL thickness map (NS, p=0.001; S, p=0.002; TS, p=0.007; NI, p=0.014; I, p=0.07; and TI, p=0.05)^(16)^, and a study in a Latino population revealed a lower mean GCL thickness in diabetic individuals without retinopathy compared with healthy controls^(17)^. Importantly, none of these studies evaluated the relationship between total macular thickness and GCL thickness.

In the literature, GCL thinning is occasionally regarded as a surrogate of neurodegeneration. Because the GCL is a component of the retina, it might be thin in eyes with thin retinas. In a study of a series of type 1 diabetic patients, a 5-µm thinner mean pericentral macular thickness was observed in eyes without retinopathy compared with healthy eyes^(7)^. Moreover, the authors of a recent publication considered retinal thinning to be an early finding in diabetic retinopathy, which may appear without a relationship to vessel density^(18)^. Accordingly, we hypothesized that thinning of the macula could result in thinning of the GCL, but found no studies that assessed the relationship between these thicknesses.

A study that evaluated the repeatability of GCL thickness measurements revealed that these were less reliable in eyes with atrophy (central macular thickness <200 µm) than in eyes with normal central macular thickness (200-300 µm)^(19)^. Although the eyes in our study met the criteria used for normal central macular thickness in the prior study, our study was designed to investigate whether significant correlations were present between GCL thickness and macular thickness in fields of the OCT map with corresponding location. We found statistically significant correlations between GCL thickness and fields 2, 3, and 4 of the OCT map in non-diabetic patients. These correlations were also detected in diabetic patients without retinopathy, and an additional correlation with field 5 was detected. The correlations were near 0.5 in patients without diabetes and near 0.6 in diabetic patients without retinopathy. Although these correlations do not completely explain the thinning of the GCL in eyes with a low macular thickness, their contributions could influence the interpretation of neurodegeneration and should be considered when comparing GCL thicknesses between diabetic and non-diabetic patients.

Although the diabetic patients in our study did not have GCL thinning that qualified as preclinical neural damage, their mean inner vessel density was lower than that in non-diabetic patients. This finding is a preclinical sign of vascular damage, which OCTA can detect. Earlier studies proposed that neural retinal damage preceded clinically detectable vascular changes. In the present study, preclinical vascular alterations appeared in diabetic patients without any changes in GCL or RNFL thicknesses. The correlation between GCL thickness and inner vessel density in group 1 suggests a tissue status that results from an adequate adjacent capillary network as the superficial capillary plexus is located in the RNFL (ganglion cell axons). The loss of this correlation in group 2 could reflect a change in tissue turgor caused by reduction of parafoveal vessel density, but a specifically designed study is needed to confirm this hypothesis.

A strength of this study was that the patients were age-matched, which reduced the effect of age on GCL thickness. Additionally, data were obtained with an OCTA device that has proven reproducibility and reliability characteristics^(10^,^20)^. Although the sample size was not large, it was sufficient to detect the reduction of parafoveal vessel density in diabetic patients without retinopathy. A potential limitation of the study was that we did not evaluate other retinal layers, which could provide additional information regarding the correlation between GCL thickness and total macular thickness^(21)^. However, we used only automated measurements to reduce variability. Another limitation was the lack of a group of patients with diabetic retinopathy. Consequently, additional studies are needed to identify a cutoff point at which GCL thickness could decrease.

In conclusion, the GCL thickness in our population was not lower in diabetic patients without retinopathy than in non-diabetic patients. Furthermore, it exhibited a substantial correlation with total macular thickness.

The reduction of parafoveal vessel density in diabetic patients without retinopathy is an earlier preclinical change than the possible reduction of the GCL thickness.
